# Differences in Micronutrient Intakes of Exclusive and Partially Breastfed Indonesian Infants from Resource-Poor Households are Not Accompanied by Differences in Micronutrient Status, Morbidity, or Growth

**DOI:** 10.1093/jn/nxaa381

**Published:** 2021-01-12

**Authors:** Claudia Leong, Rosalind S Gibson, Aly Diana, Jillian J Haszard, Sofa Rahmannia, Mohammad Brachim Ansari, Lina Sofiatul Inayah, Afini Dwi Purnamasari, Lisa A Houghton

**Affiliations:** Department of Human Nutrition, University of Otago, Dunedin, New Zealand; Department of Human Nutrition, University of Otago, Dunedin, New Zealand; Department of Human Nutrition, University of Otago, Dunedin, New Zealand; Nutrition Working Group, Faculty of Medicine, Universitas Padjadjaran, Bandung, Indonesia; Department of Human Nutrition, University of Otago, Dunedin, New Zealand; Nutrition Working Group, Faculty of Medicine, Universitas Padjadjaran, Bandung, Indonesia; Faculty of Medicine, Universitas Pasundan, Bandung, Indonesia; Nutrition Working Group, Faculty of Medicine, Universitas Padjadjaran, Bandung, Indonesia; Nutrition Working Group, Faculty of Medicine, Universitas Padjadjaran, Bandung, Indonesia; Nutrition Working Group, Faculty of Medicine, Universitas Padjadjaran, Bandung, Indonesia; Department of Human Nutrition, University of Otago, Dunedin, New Zealand

**Keywords:** exclusively breastfed, partially breastfed, infant micronutrient status, infant micronutrient intakes, growth faltering

## Abstract

**Background:**

When maternal micronutrient intakes and statuses are compromised, reductions in micronutrient concentrations in neonatal stores and human milk may result in suboptimal micronutrient intakes, statuses, and functional outcomes of breastfed infants during the critical first 6-month period.

**Objectives:**

We compared the adequacy of micronutrient intakes and statuses at 2 and/or 5 months and morbidity and growth faltering at 2, 5, and 12 months in a cohort of exclusively breastfed (EBF) and partially breastfed (PBF) infants from low-resource Indonesian households.

**Methods:**

At 2 and 5 months, the breastfeeding status and human milk intake of 212 infants were determined using the deuterium oxide dose-to-mother technique, and intakes were calculated from milk micronutrient concentrations and 3-d weighed food intakes. At 5 months, five infant micronutrient biomarkers, hemoglobin, C-reactive protein, and α-1-acid-glycoprotein were measured. Infant morbidity, weight, and length were measured at 2, 5, and 12 months. Means, medians, or proportions were reported for each group and differences between groups were statistically determined.

**Results:**

Median intakes of iron, thiamin, niacin, and vitamin B-12 were higher in PBF than EBF infants at 5 months (all *P* values < 0.05), but intakes in all infants were below adequate intakes. At 5 months, anemia was <20% in both groups, although fewer PBF versus EBF infants had vitamin B-12 deficiency (11.5% vs. 28.6%, respectively; *P* = 0.011). The mean ± SD length-for-age *z*-scores for EBF versus PBF infants at 2 months were 0.7 ± 0.9 versus −0.5 ± 1.1, respectively  (*P* = 0.158), declining to −1.4 ± 0.9 versus −1.1 ± 1.2, respectively, at 12 months (*P* = 0.059). Reported morbidity rates were generally low, with no evidence of a difference between infant groups (all *P* values > 0.126).

**Conclusions:**

Irrespective of exclusive or partial breastfeeding status, micronutrient intakes of infants were low, statuses were compromised, and growth faltering during the critical 6 months period of early infancy was present. The findings highlight the importance of improving maternal nutritional statuses and evaluating their impacts on infant outcomes.

## Introduction

The WHO recommends exclusive breastfeeding (EBF) for healthy, term infants up to 6 months of age, primarily because of the strong protective effect of EBF on gastrointestinal infections with no apparent adverse effects on infant growth (1). Nevertheless, the WHO has cautioned that in resource-poor settings where maternal iron statuses and, hence, neonatal iron stores are not optimal, EBF to 6 months may lead to iron deficiency (2). In such settings, maternal status of other micronutrients besides iron may also be compromised, reducing human milk micronutrient concentrations (3). As a result, EBF infants may be at risk of having a suboptimal micronutrient status, with the potential for impaired immune competence and subsequent growth (2). However, there are few reports on both the micronutrient intakes and statuses of EBF infants from resource-poor settings during the critical 6-month postpartum period (4). Furthermore, whether the infants have been truly EBF is uncertain, because the studies are based most frequently on maternal or caregiver self-reports, which are known to be subject to recall and social desirability bias (5).

In Indonesia, a lower middle–income country, reported rates of exclusive breastfeeding for the first 6 months (40.9%) are relatively high (6). However, most of these reports have failed to confirm whether the Indonesian infants are truly EBF (7), with limited investigations of their micronutrient intakes and statuses (8, 9). In our earlier cross-sectional study of Indonesian EBF infants from resource-poor households (10), intakes of several micronutrients (e.g., iron, zinc, selenium, and B vitamins), calculated from measurements of milk micronutrient concentrations and volume, appeared low in relation to current recommendations, although no biomarkers of micronutrient status were investigated.

Many mothers also practice partial breastfeeding (PBF) in the first 6 months in Indonesia, introducing home-prepared solid foods such as low–energy density instant chicken porridge prepared from white rice and chicken flavoring, as well as commercially produced, fortified infant cereals prepared with water and other liquids, such as tea, that are not necessarily nutritionally adequate (11, 12). Hence, shortfalls in micronutrient intakes may be even greater for infants who are PBF, and partial breastfeeding is associated with higher prevalences of both micronutrient deficiencies and adverse health consequences, such as increased susceptibility to infections and poor growth (13). Certainly, in a cohort of breastfed Indonesian infants, of whom 28% and 72% were receiving complementary foods at ages 4 and 6 months, respectively, deficiencies of iron, zinc, vitamin A, selenium, and B-12 (but not vitamin D and folate) were evident at 6 months of age (14). Clearly, more research on the adequacy of micronutrient intakes and the statuses of EBF and PBF infants in Indonesia during this critical 6-month postpartum period is warranted.

Recently, we developed a new, shortened protocol utilizing the deuterium oxide dose-to-mother technique (DTM) to classify mother-infant pairs as EBF or PBF based on the intake of water by the infant from sources other than infant milk (15). Here, utilizing this shortened DTM protocol, our primary objective was to compare the adequacy of micronutrient intakes and statuses in a cohort of EBF and PBF Indonesian infants from low-resource households at 2 and 5 months in relation to the incidences of morbidity and growth faltering at 2, 5, and 12 months. We also investigated the association between the transitional period from EBF to PBF and the incidence of morbidity and growth faltering at 2, 5, and 12 months.

## Methods

### Study site and participants

The present study is part of a longitudinal field evaluation designed to validate the classification of infants as being EBF or non-EBF using the shortened DTM (15). The study was conducted in both urban (Bandung municipality) and rural (Sumedang district) areas of West Java, Indonesia, from June 2017 to January 2018. Details of the study sites have been reported previously (3). In brief, urban (*n* = 108) and rural (*n* = 104) breastfeeding mother-infant dyads were purposely recruited from local community health centers by health workers (cadres). The inclusion criteria were apparently healthy, breastfeeding infants born full term (>37 wk of gestation) and weighing ≥2500 g at birth with no evidence of chronic disease or acute malnutrition.

The recruitment size of the cohort was chosen based on the overall validation study aim (15). A required sample size of 102 breastfeeding mother-infant dyads was estimated to detect a difference of 86.6 g/d of non-human milk intake, which was used to define EBF, and SD of 152 g/d, which was shown to define the difference between EBF and PBF with 80% power and an α of 0.05 (15). The sample size was increased to 128 dyads (64 mother-infant dyads per breastfeeding group) to account for a 25% attrition rate. For the present study, the sample size is adequate to generate reliable estimates from regression models with several covariates (16).

Ethical approval was obtained from the Human Research Ethics Committee, Faculty of Medicine, Universitas Padjadjaran, Bandung, Indonesia (05/UN6.C1.3.2/KEPK/PN/2017). Informed written consent to participate was provided by the parents or guardians.

### Sociodemographic, anthropometric, and morbidity status

Interviewer-administered, pretested questionnaires were used to collect details on the maternal sociodemographic status and parity at 2 months. The maternal weight (minimal clothing) and both the infant weight (nude) and recumbent length were measured at 2 and 5 months using calibrated equipment and standardized techniques (17). The length-for-age *z*-score (LAZ), weight-for-age *z*-score (WAZ), weight-for-length *z*-score (WLZ), and BMI-for-age *z*-score (BMIZ) were calculated with WHO AnthroPlus 3.2.2 (18).

At 2 and 5 months, infant morbidity data from the prior 2 wk (fever, cough, diarrhea, vomiting, and sore throat) were obtained through personnel interviews with the mothers. Respiratory-related illnesses (cough and/or sore throat) and gastrointestinal-related illnesses (diarrhea and/or vomiting), both with and without fever, and fever alone were recorded. Diarrhea was defined as the passage of loose or liquid stools and a high stool frequency (i.e., ≥3 stools/d). Mothers also used a standardized checklist on a morbidity calendar to record any signs and symptoms experienced by their infant over a period of 14 days, and these records were used to calculate the total number of sick days for each infant.

### Measurement of human milk volume and classification of breastfeeding status

For the shortened DTM protocol, predose saliva samples were collected on Day 0 from both the mother and infant, after which the mother was given an accurately measured, oral dose of diluted deuterium oxide. A 3-postdose sample design was used, whereby postdose saliva samples were collected from the mother-infant pairs on Days 2 or 3; Days 7, 8, or 9; and Days 13 or 14 (15). The average daily milk intake was calculated with the application of a fully Bayesian framework using a gradient-based Markov chain Monte Carlo approach implemented in Stan (15).

Mother-infant dyads were classified as EBF based on their intake of water from sources other than human milk, using a cut-off of 86.6 g/d as described by Liu et al. (15). However, in cases where infants reportedly consumed foods based on their 3-d weighed food records, they were recorded as PBF. All PBF infants at 2 months were considered as PBF at 5 months, regardless of the DTM classification. Infants who were EBF at 2 months but PBF at 5 months were classified as “transition” infants.

### Collection and micronutrient analysis of human milk samples

On Day 13 or 14, morning milk samples from a full breast were collected at 2 and 5 months for subsequent micronutrient analysis, as described previously (3). Briefly, minerals (calcium, iron, and zinc) were analyzed at the Centre for Trace Element Analysis, Department of Chemistry, University of Otago, New Zealand, by inductively coupled plasma mass spectrophotometry (Agilent 7900, Agilent Technologies), while vitamins were analyzed at the USDA/Agricultural Research Service Western Human Nutrition Research Center, Davis, CA. Milk energy was calculated from the creamatocrit method (CV = 6.9%; CreamatocritPlus; EKF Diagnostics), using the equation 385.422 + (55.656 x creamatocrit value) (19).

### Assessment of complementary food intakes

Intakes of any complementary foods at 2 and 5 months were assessed from in-home weighed food records on 3 nonconsecutive days (10). In Bandung city, mothers were trained to weigh the intakes, whereas in the Sumedang district, trained community cadres weighed in-home 12-hr food intakes (0600–1800) and performed 12-hr recalls of any foods consumed over the previous 1800–0600 period to estimate the total 24-hr food intakes. Infant intakes and major food sources of energy and nutrients were calculated using a locally produced Indonesian food composition table that took into account the government mandatory fortification of all wheat flour products with 5 micronutrients (20). Complementary foods were also grouped into 5 major sources—infant formulae, infant cereals, vitamin supplements, other cereals, and fruits and vegetables—which were ranked in relation to their contribution to the total amount of complementary food fed (as percentages) at 2 and 5 months.

### Assessment of intakes from both human milk and complementary foods

The individual milk volume consumed by each infant was used to estimate the average daily micronutrient intakes from milk for EBF and PBF infants based on analyzed milk micronutrient concentrations (mg/L or μg/L). Micronutrient intakes of total riboflavin, free thiamin, niacin, and vitamins A, E, and B-6 were calculated from the corresponding measured concentrations for each vitamer as previously described (3, 10).

Median daily intakes of energy and nutrients for PBF infants were calculated by summing the average nutrient intakes from 3-d weighed records and intakes from milk. Median intakes of EBF and PBF infants at 2 and 5 months were compared with corresponding adequate intakes (AIs) for the micronutrients from the European Food Safety Authority (21). Zinc was an exception when the AI adopted by the International Zinc Nutrition Consultative Group (IZiNCG) was applied (22). Energy intake was compared to estimated energy requirements from the FAO, WHO, and United Nations University (UNU) (23), based on median weight-for-age measurements of the WHO pooled breastfed data set.

### Assessment of biomarkers of micronutrient and inflammation status

Infant anticoagulated whole blood and serum were collected at 5 months by trained phlebotomists from morning, nonfasting, venipuncture blood samples using rigorous trace element–free collection and separation procedures, as reported previously (24). The presence of symptoms of infection, time of the blood collection, and time elapsed since the last breastfeed were all recorded.

Hemoglobin was assayed by means of a complete blood count using an automated counter (Sysmex XN-1000, Sysmex Corporation). Serum ferritin, retinol-binding protein (RBP), C-reactive protein (CRP), and α-1-acid-glycoprotein (AGP) were analyzed by a combined sandwich ELISA method (24). Serum B-12 was analyzed by an automated electro chemiluminescence immunoassay using a commercial kit (Vitamin B-12 Elecsys reagent kit, Roche Diagnostics, GmbH). Serum zinc and selenium were determined by inductively coupled plasma mass spectrometry (Agilent 7500 ICP-MS; Agilent Technologies) in the Centre for Trace Element Analysis, Department of Chemistry, University of Otago, New Zealand. All the biomarker assays for the infants were analyzed at the same time as those for the mothers, as reported previously (3). Hence, the accuracy and precision have been previously reported (3).

Serum concentrations of ferritin, RBP, and selenium were adjusted for inflammation using both CRP and AGP, as well as the biomarkers reflecting inflammation and nutritional determinants of anemia (BRINDA) regression method described previously (25). Serum zinc was adjusted for the time of day and time since last feeding before the blood collections were made, and was then adjusted for inflammation (26). The criteria used to define deficiency for each biomarker were as follows: hemoglobin, <105 g/L (27) and <110 g/L; adjusted ferritin, <12 μg/L (28, 29); adjusted zinc, <9.9 μmol/L (the recommended cut-off for children >3 years of age) (22); adjusted RBP, <0.83 μmol/L (30); adjusted selenium, <0.82 μmol/L (31); and vitamin B-12, <148 *p*mol/L (32).

### Statistical analysis

All statistical analyses were carried out using Stata 16 (Stata Corp. LLC). No adjustments for multiple testing were made. Participants with both milk concentration data and biomarker data at 5 months were included in this analysis (*n* = 159). Infants were classified as either PBF or EBF at both 2 and 5 months. Infants were further categorized as either PBF at both 2 and 5 months, EBF at both 2 and 5 months, or EBF to PBF transition. Demographic differences between these 3 groups were assessed with a Fisher's exact test for categorical variables and an ANOVA F-test for continuous variables.

Medians and 25th and 75th percentiles of daily energy and nutrient intakes from milk and complementary foods were calculated for the PBF and EBF groups at both 2 and 5 months. Differences in median total intakes between the groups were assessed with a nonparametric 2-sample test on the equality of the medians with a continuity correction. Medians and nonparametric tests were used due to skewed distributions of several of the nutrients, especially in those who were PBF.

The relationships between breastfeeding status and biomarkers were assessed by calculating medians (with 25th and 75th percentiles) or percentages for each group, as appropriate (continuous or dichotomous variables, respectively). Linear or logistic regression was used to test for differences between the groups after adjustment for location (rural or urban), maternal age, and infant sex. The same approach was taken to assess the relationships between breastfeeding status and infant growth and morbidity. The difference in the number of sick days between groups was assessed with a median test due to a skewed distribution. All analyses were run separately for each infant age (2, 5, or 12 months). Mean differences (95% CI) in infant growth across infant age groups were assessed by a paired *t*-test. Post hoc analyses comparing EBF and PBF statuses of the whole sample were carried out for vitamin B-12 deficiency and growth.

## Results

Demographic characteristics of the 159 infants by breastfeeding status are presented in **Table 1**. Mothers who followed EBF at 2 months were younger (28 years vs. 31 years, respectively) and from the rural Sumedang district (50% vs. 23%, respectively) compared to mothers with PBF infants.

**TABLE 1:** Demographic characteristics by breastfeeding status group (*n* = 159)

	PBF,^1^ *n* = 26	EBF,^1^ *n* = 63	EBF to PBF transition,^1^ *n* = 70	*P* value for difference between groups^2^
Maternal age at 2 mo PP, y	31.4 ± 6.8	28.4 ± 5.5	27.6 ± 6.5	0.029
Maternal wealth index^3^	0.0 ± 1.4	0.1 ± 1.3	0.0 ± 1.3	0.868
Maternal education, *n* (%)	—	—	—	0.641
Elementary school	6 (23.1)	12 (19.1)	15 (21.4)	
Junior high school	5 (19.2)	18 (28.6)	24 (34.3)	
Senior high school and above	15 (57.7)	33 (52.4)	31 (44.3)	
Maternal height, cm	151 ± 5	151 ± 4	151 ± 6	0.881
Maternal height <145cm, *n* (%)	3 (11.5)	6 (9.5)	11 (15.7)	0.532
Rural location, *n* (%)	6 (23.1)	35 (55.6)	32 (45.7)	0.019
Infant female sex, *n* (%)	9 (34.6)	37 (58.7)	37 (52.9)	0.120
Infant birthweight,^4^ g	3231 ± 414	3127 ± 332	3097 ± 330	0.253
Primiparous, *n* (%)	7 (26.9)	19 (30.2)	21 (30.0)	0.973
Infant weight, kg				
2 mo	4.8 ± 0.7	4.9 ± 0.5	4.9 ± 0.6	0.904
5 mo	6.8 ± 0.9	6.7 ± 0.8	6.7 ± 0.8	0.788
12 mo	8.5 ± 1.1	8.3 ± 1.0	8.4 ± 1.1	0.845
Infant length, cm				
2 mo	56.5 ± 2.3	55.8 ± 1.6	56.0 ± 2.3	0.328
5 mo	64.0 ± 2.6	62.7 ± 1.9	62.8 ± 2.4	0.030
12 mo	72.3 ± 3.1	71.4 ± 2.2	71.6 ± 2.3	0.373

Values are shown as mean ± SD or frequency (%). EBF, exclusively breastfed infants; PBF, partially breastfed infants; PP, postpartum.

1 PBF is of infants at both 2 and 5 months postpartum. EBF is of infants at both 2 and 5 months postpartum. EBF to PBF transition is of infants who were exclusively breastfed at 2 months postpartum but partially breastfed at 5 months postpartum.

2 *P* value from Fisher's exact test for categorial variables and ANOVA F-test for continuous variables.

3 An asset-based wealth index derived following the Demographic Health Survey Wealth Index guidelines (33), from a principal component analysis.

4 Missing 1 infant birthweight in the PBF group and the EBF to PBF transition group.

There was no evidence of differences among EBF, PBF, or transition infants at 2 or 5 months for any of the morbidity variables measured or for most of the growth indicators. Exceptions were less negative mean WLZ (*P* = 0.013) and BMIZ (*P* = 0.053) scores for the EBF infants (WLZ: 0.2; BMIZ: −0.2) compared with the PBF infants (WLZ: −0.3; BMIZ: −0.6) at 2 months and significantly fewer EBF infants with >2 sick days compared with the PBF infants at that time (18.6% vs. 42.3%, respectively; *P* = 0.023; **Table 2**). Mean (SD) LAZ scores for EBF versus PBF infants were −0.7 (0.9) versus −0.5 (1.1), respectively, at 2 months, −0.8 (0.8) versus −0.4 (1.2), respectively, at 5 months, and −1.4 (0.8) versus −1.1 (1.2), respectively, at 12 months. In the post hoc analysis, the decline in LAZ scores between 5 and 12 months for the whole sample was statistically significant (*P* < 0.001, from a paired *t*-test). A similar decline was also seen in WAZ scores for the entire sample of infants.

**TABLE 2:** Morbidity and growth at 2, 5, and 12 months postpartum by breastfeeding status at 5 months postpartum (*n* = 159)

	Infant age, mo	PBF, *n* = 26 at 2 mo, *n* = 26 at 5 mo, and *n* = 22 at 12 mo	EBF, *n* = 133 at 2 mo, *n* = 63 at 5 mo, and *n* = 55 at 12 mo	EBF to PBF transition, *n* = 0 at 2 mo, *n* = 70 at 5 mo, and *n* = 61 at 12 mo	*P* value for difference between groups^1^
Morbidity in the past 2 wk					
Diarrhea, *n* (%)	2	3 (11.5)	8 (6.0)	—	0.191
	5	0	4 (6.4)	6 (8.6)	
	12	1 (4.5)	6 (10.9)	6 (9.8)	0.741
Gastrointestinal, *n* (%)	2	3 (11.5)	17 (12.8)	—	0.797
	5	0	7 (11.1)	11 (15.7)	
	12	2 (9.1)	10 (18.2)	14 (23.0)	0.378
Respiratory, *n* (%)	2	8 (30.8)	28 (21.1)	—	0.382
	5	10 (38.5)	18 (28.6)	23 (32.9)	0.935
	12	8 (36.4)	15 (27.3)	22 (36.1)	0.766
Fever, *n* (%)	2	7 (26.9)	22 (16.5)	—	0.220
	5	6 (23.1)	17 (27.0)	26 (37.1)	0.247
	12	9 (40.9)	10 (18.2)	34 (34.4)	0.126
Sore throat, *n* (%)	2	0	2 (1.5)	—	
	5	0	2 (3.2)	1 (1.4)	
	12	0	0	0	
Vomit, *n* (%)	2	1 (3.9)	6 (4.5)	—	0.786
	5	0	4 (6.4)	7 (10.0)	
	12	2 (9.1)	1 (1.8)	8 (13.1)	0.155
Total number of sick days	2	1 (0, 0)	0 (0, 2)	0 (0, 2)	0.165
	5	1 (0, 5)	0 (0, 4)	1 (0, 4)	0.258
	12	4 (1, 0)	2 (0, 5)	3 (1, 8)	0.189
>2 sick days, *n* (%)	2	11 (42.3)	21 (18.6)	—	0.023
	5	8 (34.8)	19 (32.8)	27 (39.1)	0.758
	12	13 (65.0)	25 (53.2)	32 (61.5)	0.892
Growth factors					
WAZ	2	−0.7 ± 1.0	−0.6 ± 0.8	—	0.584
	5	−0.6 ± 1.0	−0.5 ± 0.9	−0.6 ± 1.0	0.867
	12	−1.0 ± 1.0	−1.0 ± 1.0	−1.0 ± 1.1	0.447
WAZ (←2SD), *n* (%)	2	2 (7.7)	6 (4.5)	—	0.411
	5	2 (7.7)	3 (4.8)	4 (5.7)	0.877
	12	3 (13.6)	8 (14.3)	13 (21.3)	0.410
LAZ	2	−0.5 ± 1.1	−0.7 ± 0.9	—	0.158
	5	−0.4 ± 1.2	−0.8 ± 0.8	−0.9 ± 1.0	0.167
	12	−1.1 ± 1.2	−1.4 ± 0.8	−1.4 ± 0.9	0.167
LAZ (←2SD), *n* (%)	2	3 (11.5)	9 (6.8)	—	0.453
	5	1 (3.9)	3 (4.8)	9 (12.9)	0.174
	12	4 (18.2)	14 (25.0)	20 (32.8)	0.108
WLZ	2	−0.3 ± 0.7	0.2 ± 1.0	—	0.013
	5	−0.4 ± 0.8	0.0 ± 0.9	0.0 ± 1.1	0.481
	12	−0.5 ± 0.9	−0.4 ± 1.0	−0.4 ± 1.1	0.760
WLZ (←2SD), *n* (%)	2	0	6 (4.5)	—	
	5	0	1 (1.6)	3 (4.3)	
	12	0	1 (1.8)	5 (8.2)	
Growth velocity, mm/d	2–5	0.83 ± 0.15	0.78 ± 0.17	0.76 ± 0.16	0.489
	5–12	0.40 ± 0.07	0.39 ± 0.06	0.39 ± 0.06	0.135
BMIZ	2	−0.6 ± 0.7	−0.2 ± 0.9	—	0.053
	5	−0.5 ± 0.8	−0.1 ± 1.0	−0.2 ± 1.1	0.530
	12	−0.4 ± 0.9	−0.2 ± 1.0	−0.3 ± 1.1	0.857

Values are mean ± SD, frequency (%), or median (25th, 75th percentiles). BMIZ, BMI-for-age *z*-score; EBF, exclusively breastfed infants; LAZ, length-for-age *z*-score; PBF, partially breastfed infants; WAZ, weight-for-age *z*-score; WLZ, weight-for-length *z*-score.

1 *P* values for differences between groups were determined from linear or logistic regression models adjusted for maternal age, location (urban or rural), and infant sex. A median test was used for number of sick days. There was no adjustment for multiple tests.

Median (with 25th and 75th percentiles) daily intakes of energy and micronutrients of the infants (*n* = 159) at 2 and 5 months from human milk only (EBF infants) and milk plus complementary foods (PBF infants) are shown in **Table 3**. Median (25th and 75th percentiles) intakes of human milk for EBF versus PBF infants were 771 mL/d (664, 882) versus 450 mL/d (163, 599), respectively, at 2 months and 799 mL/d (716, 895) versus 631 mL/d (520, 737), respectively at 5 months. For both groups, median daily intakes of energy at 2 and 5 months were below the FAO/WHO/UNU estimated energy requirements, with the shortfalls in energy being most marked for the PBF group. Median intakes of most micronutrients were below the AIs for both the EBF and PBF infants at 2 and 5 months. The exceptions were intakes for vitamin A at 2 months and calcium at 2 and 5 months for the EBF group and for vitamin B-12 at 2 months and iron at 5 months for the PBF group, when all median intakes were greater than the AIs. Significant differences existed between the median intakes of energy and most of the micronutrients for the EBF and PBF groups, although the directions of the differences were not consistent and depended on the micronutrient and the breastfeeding status.

**TABLE 3:** Infant daily energy and nutrient intakes from human milk and complementary food at 2 and 5 months postpartum by breastfeeding status (*n* = 159)

		PBF, *n* = 26 at 2 mo and *n* = 96 at 5 mo			EBF, *n* = 133 at 2 mo and *n* = 63 at 5 mo		*P* value for difference between PBF vs. EBF^2^
	Infant age, mo	Human milk	CF	Total	Human milk	AI,^1^ unit/d	
Human milk, mL/d	2	450 (163, 599)	—	—	771 (664, 882)	800	<0.001
	5	631 (520, 737)	—	—	799 (716, 895)	800	<0.001
Energy,^3^ kcal/d	2	307 (111, 409)	103 (0, 253)	403 (368, 485)	527 (454, 603)	544	<0.001
	5	394 (324, 460)	0 (0, 95)	448 (383, 522)	498 (447, 559)	590	0.044
Calcium, mg/d	2	115 (40, 168)	59 (0, 144)	185 (165, 240)	212 (182, 258)	200	0.143
	5	162 (126, 202)	0 (0, 39)	186 (149, 236)	203 (167, 244)	200	0.698
Iron, mg/d	2	0.10 (0.04, 0.17)	1.14 (0.00, 2.72)	1.19 (0.34, 2.72)	0.22 (0.16, 0.31)	0.3	<0.001
	5	0.11 (0.07, 0.17)	0.00 (0.00, 1.59)	0.26 (0.12, 1.64)	0.14 (0.11, 0.21)	0.3	0.001
Zinc, mg/d	2	0.58 (0.14, 0.95)	0.89 (0.00, 1.68)	1.44 (1.07, 2.27)	1.01 (0.66, 1.39)	2	0.005
	5	0.48 (0.26, 0.72)	0.00 (0.00, 0.83)	0.78 (0.44, 1.55)	0.66 (0.50, 0.99)	2	0.559
Vitamin A, RAE/d	2	273 (68, 376)	92 (0, 203)	331 (277, 507)	497 (333, 747)	350	0.058
	5	236 (148, 356)	0 (0, 54)	286 (205, 397)	292 (201, 390)	350	0.698
Total thiamin (B-1),^4^ mg/d	2	0.04 (0.01, 0.06)	0.06 (0.00, 0.13)	0.10 (0.06, 0.18)	0.08 (0.06, 0.09)	0.2	0.268
	5	0.06 (0.04, 0.09)	0.00 (0.00, 0.06)	0.09 (0.06, 0.14)	0.08 (0.06, 0.09)	0.2	0.027
Total riboflavin (B-2),^5^ mg/d	2	0.04 (0.01, 0.06)	0.15 (0.00, 0.30)	0.19 (0.07, 0.33)	0.08 (0.06, 0.10)	0.3	0.017
	5	0.07 (0.04, 0.09)	0.00 (0.00, 0.08)	0.10 (0.07, 0.17)	0.09 (0.06, 0.13)	0.3	0.119
Total niacin (B-3),^6^ mg/d	2	0.33 (0.16, 0.71)	0.25 (0.00, 0.74)	0.91 (0.52, 1.46)	0.69 (0.45, 1.07)	2	0.498
	5	0.35 (0.23, 0.52)	0.00 (0.00, 0.20)	0.53 (0.34, 0.87)	0.37 (0.29, 0.49)	2	<0.001
Vitamin B-6^7^ mg/d	2	0.04 (0.01, 0.06)	0.02 (0.00, 0.06)	0.07 (0.05, 0.13)	0.06 (0.04, 0.09)	0.1	0.498
	5	0.06 (0.03, 0.09)	0.00 (0.00, 0.02)	0.07 (0.04, 0.11)	0.08 (0.06, 0.12)	0.1	0.476
Vitamin B-12, μg/d	2	0.11 (0.03, 0.17)	0.25 (0.00, 0.52)	0.43 (0.23, 0.62)	0.22 (0.18, 0.29)	0.4	0.005
	5	0.17 (0.14, 0.24)	0.00 (0.00, 1.55)	0.25 (0.17, 1.83)	0.21 (0.18, 0.26)	0.4	0.011

Values are medians (25th, 75th percentiles). AI, adequate intake; CF, complementary food; EBF, exclusively breastfed infants; EFSA, European Food Safety Authority; PBF, partially breastfed infants; RAE, retinol activity equivalents.

1 Adequate food intakes in infants aged 0 to 6 months, with values obtained from the EFSA (21), except for zinc, which had values obtained from the International Zinc Nutrition Consultative Group (22), and estimated energy requirements, which had values obtained from the FAO, WHO, and United Nations University (23), based on the median weight for age of the WHO pooled breastfed data set.

2 *P* values for differences between PBF and EBF were calculated using a nonparametric 2-sample test on the equality of the medians with a continuity correction. No adjustments were made for multiple testing.

3 Milk energy was based on the creamatocrit method, using the equation 385.422 + (55.656 x Creamatocrit value) (19).

4 Vitamin B-1 from human milk was calculated based on the measured concentrations for each vitamer and expressed as free thiamin [thiamin + (thiamin pyrophosphate × 0.707) + (thiamin monophosphate × 0.871)].

5 Vitamin B-2 from human milk was calculated based on the measured concentrations for each vitamer and expressed as riboflavin [riboflavin + (flavin adenine dinucleotide × 0.479) + (flavin mononucleotide × 0.825)].

6 Vitamin B-3 from human milk was calculated as nicotinamide [nicotinamide + (nicotinamide adenine dinucleotide × 0.184)].

7 Vitamin B-6 from human milk was calculated as (pyridoxal + pyridoxine).

For the PBF infants at 2 months, infant formula was the major source of complementary food, and hence the major contributor of energy, iron, zinc, and vitamins A, B-6, and B-12 from complementary foods (**Supplementary Table 1**). At 5 months of age, however, infant cereals replaced infant formula as both the major complementary food consumed and the major source of most micronutrients from complementary food, with the exception of vitamin A and energy, for which infant formula remained the main source from complementary foods.

At 5 months of age, there were no significant differences among the EBF, PBF, or transition groups for the median micronutrient concentrations for the 5 biomarkers or for the prevalences of anemia or micronutrient deficiencies, with the exception of serum B-12 deficiency (**Table 4**). EBF infants had significantly lower serum B-12 concentrations and a higher prevalence of B-12 deficiency compared to PBF and transition infants. In the post hoc analysis, for the entire sample of infants at 5 months, PBF infants had a significantly lower prevalence of vitamin B-12 deficiency than EBF infants (11.5% vs. 28.6%, respectively; *P* = 0.011).

**TABLE 4:** Biomarkers at 5 months postpartum by breastfeeding status at 5 months (*n* = 159)

	PBF, *n* = 26 at 5 mo	EBF, *n* = 63 at 5 mo	EBF to PBF transition, *n* = 70 at 5 mo	*P* value for difference between groups^1^
Hemoglobin,^2^ g/L	116 (107, 126)	114 (110, 121)	114 (108, 121)	0.553
<110 g/L, *n* (%)	11 (42.3)	15 (23.8)	24 (34.8)	0.276
<105 g/L, *n* (%)	4 (15.4)	9 (14.3)	8 (11.6)	0.899
Iron serum ferritin,^3^ μg/L	32 (17, 82)	37 (23, 59)	34 (17, 72)	0.950
<12 μg/L, *n* (%)	4 (15.4)	6 (9.5)	9 (12.9)	0.835
IDA, *n* (%)	2 (7.7)	2 (3.2)	7 (10.1)	0.497
Serum zinc,^3^ μmol/L	11.1 (10.4, 14.3)	11.3 (10.2, 12.5)	10.9 (9.5, 12.5)	0.375
<9.9 μmol/L, *n* (%)	5 (21.7)	10 (17.9)	18 (32.1)	0.233
Serum RBP, μmol/L	1.0 (0.8, 1.1)	1.0 (0.9, 1.0)	0.9 (0.8, 1.1)	0.925
<0.83 μmol/L, *n* (%)	8 (30.8)	12 (19.1)	22 (31.4)	0.313
Serum selenium,^3^ μmol/L	0.78 (0.62, 0.92)	0.81 (0.72, 0.87)	0.75 (0.68, 0.88)	0.227
<0.82 μmol/L, *n* (%)	14 (60.9)	30 (53.6)	37 (66.1)	0.256
Serum B-12,^3^ *p*mol/L	396 (173, 555)	224 (142, 296)	242 (196, 308)	<0.001
<148 *p*mol/L, *n* (%)	2 (8.7)	16 (28.6)	7 (12.7)	0.037
CRP, mg/L	0.57 (0.26, 0.98)	0.48 (0.21, 1.20)	0.47 (0.19, 1.57)	0.946
>5 mg/L, *n* (%)	2 (7.7)	7 (11.1)	9 (12.9)	0.830
AGP, g/L	0.38 (0.26, 0.74)	0.48 (0.35, 0.84)	0.58 (0.34, 0.83)	0.305
>1 g/L, *n* (%)	0	11 (17.5)	11 (15.7)	

Values are shown as a median (25th, 75th percentiles) or frequency (%). The criteria used to define deficiency for each biomarker were as follows: hemoglobin, <105 g/L (27) and <110 g/L; adjusted ferritin, <12 μg/L (28, 29); adjusted zinc, <9.9 μmol/L (22); adjusted RBP, <0.83 μmol/L (30); adjusted selenium, <0.82 μmol/L (31); and vitamin B-12, <148 *p*mol/L (32). For zinc: adjusted for time of the day and interval since the last meal = exp {unadjusted in biomarkers + [regression coefficient for time of day x (time of day) – (regression coefficient for interval since previous meal x interval since previous meal)]}. For ferritin, RBP, zinc, and selenium: adjusted for inflammation = exp {unadjusted in biomarkers – (regression coefficient for CRP) x [CRP − (maximum of lowest decile for CRP)] − (regression coefficient for AGP) x [AGP – (maximum of lowest decile for AGP)]}. AGP, α-1-acid-glycoprotein; CRP, C-reactive protein; EBF, exclusively breastfed infants; IDA, iron deficiency anemia; PBF, partially breastfed infants; RBP, retinol binding protein.

1 *P* values for differences between groups were determined from linear or logistic regression models adjusted for maternal age, location (urban or rural), and infant sex. No adjustments were made for multiple tests.

2 Missing 1 hemoglobin in the EBF to PBF transition group.

3 Missing data on zinc in 24 records; selenium in 3 PBF, 7 EBF, and 14 EBF to PBF transition group records; 25 B-12 in 3 PBF, 7 EBF, and 15 EBF to PBF transition group records.

## Discussion

Our simultaneous measurements of the volume and micronutrient composition of milk and the weighed intakes of complementary foods generated accurate data on the micronutrient intakes of Indonesian infants from low-resource households who were truly EBF or PBF at 2 and 5 months. For both groups, the median intakes for almost all micronutrients were below the AIs at 2 and/or 5 months of age, which were accompanied at 5 months of age by risks of deficiency for all the 5 micronutrient biomarkers measured. However, because the AIs are based on the average intake of each micronutrient from human milk, they cannot be used to estimate the prevalences of inadequate intakes. Furthermore, a single average value for the transfer of each micronutrient in mature human milk has been assumed for the AI estimates, even though concentrations for almost all micronutrients decline between 2 and 5 months, as noted earlier (3). This decline is especially marked for human milk zinc concentrations and accounts for the seemingly greater deficit in the median zinc intakes at 5 months in relation to the AI. Growth faltering was apparent at 2 and 5 months, irrespective of breastfeeding status, with a marked deterioration in linear and ponderal growth by 12 months in all 3 groups (EBF, PBF, and transition).

### Adequacy of micronutrient intakes and status by breastfeeding status

The consistently higher intakes of iron and zinc for the infants categorized as PBF are not unexpected given the lower concentrations of iron and zinc in human milk (34) compared with the levels in both the infant formula and infant cereals consumed by the PBF infants (11, 35, 36). Nevertheless, despite the higher intakes of iron and zinc, there was no evidence of differences in the risks of deficiency of iron (based on hemoglobin and adjusted serum ferritin) and zinc (based on adjusted serum zinc) at 5 months among the 3 groups. This inconsistency is probably associated with the higher bioavailability of both iron and zinc from human milk (36, 37) compared with cow's milk–based infant formula (36, 38) and fortified infant cereal (39, 40). Additional contributing factors may include the limited capacity of such young infants to regulate iron homeostasis (41) and the insensitivity of serum zinc to changes in dietary zinc intakes (42).

Of interest is the greater risk for low concentrations of serum zinc compared to ferritin in this infant cohort at 5 months of age, irrespective of breastfeeding status. Several factors may account for the lower risk of iron deficiency compared to zinc deficiency among the infants at 5 months. Few of our mothers at 5 months had low body iron stores (BIS; i.e., 15%) (3), which suggests that their BIS during pregnancy were likely to be adequate. This finding suggests that our infants were probably endowed with a store of iron at birth (35) which, when augmented with the contribution of iron from human milk, ensured that the iron requirements of even the EBF infants could be met at 5 months of age, with the PBF infants receiving additional sources of iron from infant formulae and infant cereals.

In contrast, two-thirds of our lactating mothers had serum zinc concentrations at 5 months below the reference cut-off used (3), suggesting that their zinc status during pregnancy, unlike their iron status, was also likely to have been poor. As a result, it is conceivable that the amount of hepatic zinc accrued in utero (43) and reportedly mobilized during early infancy to supplement the infant's needs for zinc was reduced. Furthermore, irrespective of breastfeeding status, the zinc intakes of the infants on average fell short of the AI adopted by IZiNCG (22), and at 5 months the prevalence of serum zinc levels less than the reference cut-off applied was relatively high for all 3 infant groups (i.e., ≥18%). A coexisting low selenium status among both the EBF and PBF infants may be an additional factor negatively impacting the infant zinc status. Selenium is said to interact with certain zinc finger proteins involved in DNA repair (44). Finally, our infants were probably exposed to pervasive fecal contamination via multiple and interrelated fecal-oral pathways (45). Chronic, subclinical exposure to fecal pathogens has been strongly linked to environmental enteric dysfunction (EED) (46), which is known to impair absorption and increase intestinal endogenous losses of zinc (47, 48). In contrast, an inhibitory effect of iron on zinc absorption and/or metabolism is unlikely to be a contributing factor, as the only source of dietary iron at 5 months for the EBF infants was milk, whereas for the PBF infants, the major source of iron was iron-fortified foods (49, 50).

The low selenium status of both EBF and PBF infants observed here was consistent with our earlier report on Sumedang infants (24) and was attributed to low regional soil selenium levels (51). As a consequence, selenium concentrations in milk (3) and probably in any locally grown foods used for complementary feeding are also likely to be relatively low. This suggestion is supported by the low intake of selenium for the EBF infants supplied by milk (i.e., 9.4 μg/day) and by the finding that more than 50% of EBF infants had serum selenium levels below the concentration of optimal activity of ≥1 of the selenoproteins (31). We have no data on selenium intakes for the PBF or transition infants because of the lack of selenium food composition values, but a high proportion (at least 60%) of these infants appeared at risk for a low selenium status at 5 months.

Supplementing vitamin A during the early postpartum period (i.e., the first 40 days) is regularly carried out in Indonesia in view of the high risk of infants being born with low vitamin A reserves. Indonesian pregnant women are often at risk of vitamin A deficiency due to the combined effect of the high demands for vitamin A in the latter half of pregnancy and inadequate dietary intakes (52). In our earlier report on the lactating mothers of these infants (3), a third had low serum RBP levels at 5 months, which were associated with retinol concentrations in milk that were markedly lower at 5 months compared to 2 months (3), despite receiving vitamin A supplements at 40 days postpartum. Together, these trends emphasize that the effect of postpartum vitamin A supplements is transient (53) and explain the higher vitamin A intakes in the EBF than PBF infants at 2 months and the subsequent marked decline in intakes of vitamin A at 5 months. The large average shortfalls in vitamin A intakes at 5 months in both the EBF and PBF infants were accompanied by a high prevalence of low serum RBP concentrations among the infants, perhaps exacerbated by higher utilization rates imposed by frequent infections (54). It is conceivable that the low zinc statuses of these infants may have also contributed to the low infant serum RBP concentrations, as zinc plays a role in the hepatic synthesis of RBP, and thus in the transport and tissue utilization of vitamin A (54, 55).

The persistently low intakes of almost all the B vitamins in both the EBF and PBF infants reflected, in part, their low concentrations in milk, arising from inadequacies in maternal diets reported earlier (3). Deficits in B-vitamin intakes in both maternal and infant diets have been reported by others in Indonesia (10, 11, 20) and elsewhere in Asia (56–58), which in some cases have been associated with maternal (59–61) and/or infant deficiencies (14). Here, only the infant vitamin B-12 status was assessed at 5 months; not surprisingly, the EBF infants were the group with the highest risk of B-12 deficiency in view of the low B-12 milk concentrations of their mothers, as noted previously (3). Hence, the infants had low B-12 intakes at both 2 and 5 months of age. These findings emphasize the importance of adequate sources of B-12 in maternal diets to ensure optimal B-12 concentrations in milk (62, 63).

### Morbidity and growth outcomes

We found no apparent evidence that our EBF infants experienced less morbidity from gastrointestinal or other comorbidities at 2 and 5 months than PBF or transition infants at 5 months. Nevertheless, fewer EBF infants had >2 sick days (*P* = 0.023) at 2 months.

Similarly, we observed no meaningful differences in linear growth among the 3 groups, with growth faltering being most severe for all infants at 12 months of age. The lower WLZ and BMIZ scores of the PBF infants as compared to the EBF infants at 2 months of age were associated, at least in part, with their significantly lower energy intakes at that time, which persisted through to 5 months of age. Deficits in energy intake were also observed in our earlier Indonesian study of infants at 6, 9, and 12 months of age, although these estimates were based on the energy contributions from complementary foods alone (11). Several other investigators have reported inadequacies in energy and micronutrient intakes from complementary foods among Indonesian infants when compared to their WHO estimated needs (64).

Several factors probably contributed to the absence of a protective effect of EBF on gastrointestinal infections, other comorbidities, or linear growth faltering (65). First, all our infants were from low-resource households with median intakes of almost all micronutrients below their respective AI, and these shortfalls led to deficiencies of selenium, zinc, iron, and vitamins A and B-12 at 5 months across the 3 infant groups. Second, as most of these micronutrients (except B-12) have a key role in immune functions (66), such deficiencies were likely to compromise the infant's immune response, leading to increased susceptibility to infectious episodes in all 3 groups. The stimulation of the body's immune/inflammatory response triggered by infections can also impair linear growth, although the magnitude of the effect depends on the pathogens involved (67). The mechanisms may include reductions in intake, digestion, and absorption of nutrients and accelerated nutrient losses, all accompanied by increases in caloric requirements (66, 68). These adverse metabolic effects can occur even in the absence of overt clinical symptoms of disease (69). Our relatively low prevalence of elevated levels of serum CRP, an acute phase reactant, suggests that the infections experienced by all 3 groups of infants here were of a low intensity (66).

Third, there was no evidence of differences in the prevalences of deficiency for the 2 potentially growth-limiting micronutrients—zinc and vitamin A—by breastfeeding status. However, vitamin A deficiency does not appear to be growth limiting unless the vitamin A deficiency state is severe (70). Here, instead of serum retinol as a biomarker of vitamin A status, we used serum RBP, as it is more stable and easier to analyze (71). Concentrations of RBP in all 3 groups at 5 months were indicative of moderate but not severe vitamin A deficiency. This finding suggests that zinc deficiency, not vitamin A deficiency, is most likely contributing, at least in part, to the poor linear growth observed here. Zinc is involved in multiple metabolic pathways and is required for cell division and protein and DNA synthesis (72). Fourth, because these infants were probably exposed to fecal contamination, growth faltering may have been exacerbated by the multiple adverse effects of EED described earlier (73). Indeed, some estimates suggest that EED explains a higher percentage of growth faltering than diarrhea (74). Finally, we recognize that even at 2 months of age, there was evidence of linear growth faltering in both our EBF and PBF groups, which may have been associated with prenatal or early postnatal exposures not measured here. Indeed, an earlier study in Indonesia concluded that linear growth among rural infants in West Java was determined more by the prenatal environment than by postnatal factors (75).

## Advantages and limitations

We believe our study had many strengths. Unlike our earlier cross-sectional study (10), this study was longitudinal, with low attrition, and our assessment of the adequacy of intakes of micronutrients was based on measurements of both volumes of human milk intake via DTM and micronutrient compositions of milk, together with weighed intakes of complementary foods. With the use of DTM, we were able to accurately categorize infant breastfeeding status (EBF and PBF) and to distinguish those who transitioned from EBF at 2 months to PBF thereafter, creating a group which has been rarely investigated elsewhere. However, it should be noted that the classification of breastfeeding status by the DTM provides an accurate categorization of infants by breastfeeding status over a 2-wk period (i.e., 14-d postdose sampling timeframe) only. The consumption of any of food or drink prior to this testing period would not be detected.

We also only assessed infant biomarker status with 5 biomarkers at 1 time point (i.e., 5 months postpartum), despite the apparent inadequacies in intake of almost all the micronutrients, especially the B-vitamins, such as thiamin, riboflavin, and niacin, at 2 and 5 months. Moreover, the lack of reference cut-offs for serum zinc during infancy makes it difficult to interpret the risk of zinc deficiency, although there were no differences in risks among the 3 infant groups. In addition, our complementary food intakes were restricted to 3 days and, because of intermittent feeding, did not capture all the complementary foods or beverages fed during the 14-d DTM period, accounting for the seemingly 0 intake of micronutrients from complementary foods for the PBF group at 5 months. Moreover, because our average intakes were compared against AIs, we could not estimate the prevalences of inadequate intakes of micronutrients for the infants at 2 and 5 months. In addition, our morbidity data may be prone to measurement error because they were based on maternal recall over the prior 2 wk only, with no confirmation by a medical professional. Moreover, the incidences of morbidity from in the prior 2 wk at 2, 5, and 12 months may have been too low to observe any differences among the 3 infant groups. Finally, our observational analysis precludes us from making any causal inferences, and our opportunistic recruitment prevents our results from being generalized to other infants from resource-poor households in Indonesia. However, our reported data for diarrhea and fever were relatively comparable to those reported for infants aged <6 months (i.e., 8.3% and 20.1%, respectively, in a recent national survey in 2017) (76), although fewer infants studied here were stunted compared to the national average (23.1% for infants 0–5 months) (77). Nevertheless, by applying state-of-the-art techniques, we believe our study contributes to the limited comparative data on the adequacy of micronutrient intakes and statuses of infants less than 6 months of age who have been accurately categorized as EBF or PBF.

## Conclusion

Even though dietary intakes of PBF infants were significantly higher than those of EBF infants for most micronutrients, average intakes were below the AIs for both groups. Moreover, these differences in micronutrient intakes were not accompanied by any differences in the infant micronutrient biomarkers analyzed at 5 months, nor morbidity or growth outcomes through to 12 months of age. Finally, our findings of suboptimal dietary intakes and statuses among these infants in the first 6 months of life, irrespective of feeding, together with evidence of growth faltering, indicate that future studies should focus on strategies to improve maternal statuses during lactation and should evaluate their impacts on infant outcomes.

## Supplementary Material

nxaa381_Supplemental_File

## Notes

This work was supported by the Bill & Melinda Gates Foundation, Seattle, WA, USA.

Author disclosures: The authors report no conflicts of interest.

Supplemental Table 1 is available from the “Supplementary data” link in the online posting of the article and from the same link in the table of contents at http://academic.oup.com/jn.

Abbreviations used: AGP, α-1-acid-glycoprotein; AI, adequate intake; BIS, body iron stores; BMIZ, BMI-for-age *z*-score; CRP, C-reactive protein; DTM, deuterium oxide dose-to-mother technique; EBF, exclusively breastfed; EED, environmental enteric dysfunction; IZiNCG, International Zinc Nutrition Consultative Group; LAZ, length-for-age *z*-score; PBF, partially breastfed; RBP, retinol-binding protein; UNU, United Nations University; WAZ, weight-for-age *z*-score; WLZ, weight-for-length *z*-score.
