# Supplementary material for: Differences in Micronutrient Intakes of Exclusive and Partially Breastfed Indonesian Infants from Resource-Poor Households are Not Accompanied by Differences in Micronutrient Status, Morbidity, or Growth
Source: J Nutr. 2021 Jan 12;151(3):705–15. doi: 10.1093/jn/nxaa381 (PMC7948196; doi:10.1093/jn/nxaa381)
Supplement: nxaa381_Supplemental_File [file nxaa381_supplemental_file.docx]

**Supplementary Table 1** Complementary food sources of nutrients of partially breastfed infants at 2 and 5 mo postpartum^1^

|  | 2 mo (n = 17 with food eaten) *61% of PBF* | | |  | 5 mo (n = 42 with food eaten) *37% of PBF* | | |
| --- | --- | --- | --- | --- | --- | --- | --- |
| Nutrient | Rank | Food group | % of total CF |  | Rank | Food group | % of total CF |
| Energy | 1 | Infant formula | 99.7 |  | 1 | Infant formula | 64.1 |
|  | 2 | Infant cereal | 0.3 |  | 2 | Infant cereal | 24.8 |
|  |  |  |  |  | 3 | Other cereals | 9.2 |
|  |  |  |  |  | 4 | Fruits and vegetables | 1.8 |
| Iron | 1 | Infant formula | 92.6 |  | 1 | Infant cereal | 49.7 |
|  | 2 | Supplement - vitamins | 6.5 |  | 2 | Infant formula | 47.8 |
|  | 3 | Infant cereal | 0.3 |  | 3 | Other cereals | 2.0 |
|  |  |  |  |  | 4 | Fruits and vegetables | 0.5 |
| Zinc | 1 | Infant formula | 99.3 |  | 1 | Infant cereal | 62.1 |
|  | 2 | Infant cereal | 0.7 |  | 2 | Infant formula | 33.0 |
|  |  |  |  |  | 3 | Other cereals | 4.3 |
|  |  |  |  |  | 4 | Fruits and vegetables | 0.5 |
| Vitamin A RAE | 1 | Infant formula | 83.2 |  | 1 | Infant formula | 73.2 |
|  | 2 | Supplement - vitamins | 16.6 |  | 2 | Infant cereals | 20.7 |
|  | 3 | Infant cereal | 0.2 |  | 3 | Supplement - vitamins | 4.5 |
|  |  |  |  |  | 4 | Fruits and vegetables | 1.3 |
|  |  |  |  |  | 5 | Other cereals | 0.2 |
| Vitamin B-6 | 1 | Infant formula | 78.0 |  | 1 | Infant cereal | 54.2 |
|  | 2 | Supplement - vitamins | 22.0 |  | 2 | Infant formula | 26.3 |
|  |  |  |  |  | 3 | Other cereals | 8.4 |
|  |  |  |  |  | 4 | Supplement - vitamins | 5.6 |
|  |  |  |  |  | 5 | Fruits and vegetables | 5.4 |
| Vitamin B-12 | 1 | Infant formula | 87.1 |  | 1 | Infant cereal | 96.0 |
|  | 2 | Infant cereal | 9.8 |  | 2 | Infant formula | 3.9 |
|  | 3 | Supplement - vitamins | 3.1 |  |  |  |  |

Partially breastfed, PBF; exclusively breastfed, EBF; retinol activity equivalents, RAE.

^1^ Complementary foods were also grouped into five major sources - infant formulae, infant cereals, vitamin supplements, other cereals, fruits and vegetables, which were ranked in relation to their contribution to the total amount of complementary food fed (as %) at 2 and 5 mo postpartum.
